# Sampling method for surveying complex and multi-institutional partnerships: lessons from the Global Polio Eradication Initiative

**DOI:** 10.1186/s12889-020-08592-x

**Published:** 2020-08-12

**Authors:** Michael A. Peters, Wakgari Deressa, Malabika Sarker, Neeraj Sharma, Eme Owoaje, Riris Andono Ahmad, Tawab Saljuqi, Eric Mafuta, Olakunle Alonge

**Affiliations:** 1 Johns Hopkins University Bloomberg School of Public Health, Baltimore, MD USA; 2 Addis Ababa University School of Public Health, Addis Ababa, Ethiopia; 3 BRAC University JP Grant School of Public Health, Dhaka, Bangladesh; 4 Indian Institute of Health Management Research, Jaipur, India; 5 University of Ibadan College of Medicine, Ibadan, Nigeria; 6 Gadjah Mada University Faculty of Medicine, Yogyakarta, Indonesia; 7 Global Innovations Consultancy Services, Kabul, Afghanistan; 8 University of Kinshasa School of Public Health, Kinshasa, Democratic Republic of the Congo

**Keywords:** Complex initiative, Evaluation, GPEI, Lessons learned, Polio, Sampling

## Abstract

**Background:**

Complex global initiatives, like the Global Polio Eradication Initiative (GPEI), have prevented millions of paralyses and improved the health status of diverse populations. Despite the logistical challenges these initiatives must overcome at several levels, scant methods exist for systematically identifying and reaching a range of actors involved in their implementation. As a result, efforts to document the lessons learned from such initiatives are often incomplete. This paper describes the development and application of the Synthesis and Translation of Research and Innovations from Polio Eradication (STRIPE) systematic approach for identifying a comprehensive sample of actors involved in the GPEI.

**Results:**

The survey for collecting lessons learned from the GPEI was conducted at the global level and within seven countries that represented GPEI operational contexts. Standard organizational and operational levels, as well as goals of program activities, were defined across contexts. Each survey iteration followed similar methodologies to theorize a target population or “universe” of all polio-related actors in the study area, enumerate a source population of specific individuals within the target population, and administer the survey to individuals within the source population. Based on the systematic approach used to obtain a comprehensive sample for lessons learned in GPEI, steps for obtaining a comprehensive sample for studying complex initiatives can be summarized as follows: (i) State research goal(s); (ii) Describe the program of interest; (iii) Define a sampling universe to meet these criteria; (iv) Estimate the size of the sampling universe; (v) Enumerate a source population within the universe that can be feasibly reached for sampling; (vi) Sample from the source population; and (vii) Reflect on the process to determine strength of inferences drawn.

**Conclusions:**

The application of these methods can inform future evaluations of complex public health initiatives, resulting in better adoption of lessons learned, ultimately improving efficacy and efficiency, and resulting in significant health gains. Their use to administer the STRIPE lessons learned survey reflects experiences related to implementation challenges and strategies used to overcome barriers from actors across an extensive range of organizational, programming, and contextual settings.

## Background

The global health landscape contains a multitude of complex initiatives, defined as those having “multiple activity components; varied settings for implementation of different sets of activities; systems-strengthening efforts; capacity building; efforts to influence policy changes; use of health diplomacy to achieve the aims of the initiative; and implementation at multiple levels through a large number of diverse, multisectoral partners at the country level” [1]. Some examples include vertical and disease-focused initiatives [e.g. Global Fund to Fight AIDS, Tuberculosis and Malaria; Global Polio Eradication Initiative (GPEI); US President’s Emergency Plan for AIDS Relief (PEPFAR); President’s Malaria Initiative (PMI)]; programs that focus on a certain aspect of health services delivery (e.g. vaccine support provided by Gavi, the Vaccine Alliance); and broader health systems strengthening (e.g. research grants provided by the Alliance for Health Policy and Systems Research).

Although much attention has been paid to the evaluation of these complex initiatives, few methods exist to systematically identify and select comprehensive samples of populations involved in their implementation [1, 2]. Such methods would facilitate the identification of lessons that may be applicable for improving efficacy within the initiative, in addition to generating lessons that can be applied to other programs. It is essential that evaluations are done to describe these lessons about the processes, implementation strategies, and experiences of individuals at various operational levels and across contexts within these initiatives, and that methods are developed to ensure that these lessons can be generalized and appropriately incorporated in future efforts [3]. While many studies seek to distill these lessons, few adopt a systematic approach to ensure that perspectives across the spectrum of involved actors are captured, instead relying primarily on the experiences of leadership and a few key actors. Without a systematic approach, the valuable tacit knowledge of those involved with program implementation may not be captured, in which case lessons learned will not realize their full potential to improve future efforts.

In the epidemiological tradition, a key principle in generalizing findings of a study to a larger group is to define a target, a source, and a study population (See Table 1). Aspects of this principle can be extended to describe overall experiences and lessons learned within the population of actors involved in a complex initiative. However, unlike populations in epidemiology, which are often defined by characteristics through which individuals can be readily recruited into a study (e.g. gender, age, exposure to specific risk factors), populations of actors involved in a complex and multi-institutional initiative may be hard to identify (that is, hard-to-reach) due to lack of an easily identified characteristic that is shared by all those necessary to make an initiative successful. For example, the GPEI - which has prevented millions of paralyses from the poliovirus and improved the health status of diverse populations [5] - is a partnership comprised of national governments and five^1^ international organizations, thus there is no designation such as being a GPEI “staff-member” which would identify membership in polio eradication at global or national levels. Furthermore, challenges like language diversity, documentation problems, lack of internet connectivity, and time since involvement in polio activities present additional barriers to collecting information from all those involved in polio eradication.

**Table 1:** Definitions of population groups

The definition of target, source, and study populations are interrelated and are a starting point for the methods described in this paper. These concepts are defined as follows [4]; • Target Population: The collection of individuals, items, measurements, etc., about which inferences are desired. The term is sometimes used to indicate the population or group from which a sample or study population is drawn and sometimes to denote a reference population about which inferences are desired • Source Population: The group from which a study group is selected • Study Population: The group selected for investigation.	

Social sciences approaches used for generalizing findings of a study rely on sampling from a universe of population groups identified by fairly overt and homogenous characteristics, similar to the epidemiological traditions (see Table 1). The universe “consists of all survey elements that qualify for inclusion in the research study, and the precise definition of the universe for a particular study is set by the research question, which specifies who or what is of interest” [6]. Roughly analogous to the target population in epidemiology, the question-based nature of a ‘universe’ allows for more flexibility in defining who can exist within the qualifying population. A discussion of sampling within the field of organizational research studies noted the reluctance of authors to explicitly state the universe, or target population to which their findings are generalizable [7]. Other methods have been developed to obtain sampling frames (source populations) for organizational research, but these methods rely on the presence of a variety of well-maintained centralized lists, such as unemployment insurance databases that do not exist in the context of polio eradication [8].

Approaches have also been developed for sampling hidden populations in public health initiatives, such as men who have sex with men [9], drug users [10], and undocumented immigrants [11], and aspects of these approaches can be adapted for complex global health initiatives involving multiple actors. Although these techniques yield valuable lessons, when target and source populations are not well described and identified, as seen with complex global health initiatives like the GPEI, findings can be based on samples that lack representativeness, and may miss important lessons learned at various levels of operation. Other methods have been developed to study complex interventions and systems, including system dynamic modelling, agent-based modelling, network analysis, and systems mapping [12]. These methods are useful in understanding how interventions and systems interact with each other, describing how individuals/agents behave within complex systems, and/or hypothesizing and modeling intervention impacts. Although expanding in applicability, these methods are still under-developed in their current form to guide sampling from complex systems which have a wide breadth of actors working at various levels. A review of selected studies of complex initiatives conducted between 2007 and 2011 demonstrates the lack of attempts to quantitatively describe target and source populations (Table 2).

**Table 2:** Review of descriptions of target, source, and study populations in previous studies of complex initiatives

Initiative/ Study	Objective of Study	Target Population	Source Population	Study Population
External Evaluation Of The President’s Malaria Initiative (PMI) [13]	Review the first five years (FY06-FY10) of PMI’s activities, organized around six core objectives	Not described	Focus on key actors, both internal and external to PMI. Not quantitatively described 5/15 focus countries visited	65 respondents with described position/role
Five Year Evaluation of the Global Fund: Study Area 1; Organizational Development study [14]	Assess organizational efficiency and effectiveness of the Global Fund,	Described staff in terms of unit (7 types) and level (3; senior managers, managers, and other staff). Total staff estimates were not quantitatively described	283 Secretariat staff at the time of evaluation	89 Secretariat staff were involved (33 in pre-assessment meetings that identified 56 additional respondents)
Five Year Evaluation of the Global Fund: Study Area 2 [15]	Assess effectiveness of the Global Fund partnership environment	Not described	Partners and stakeholders at the country and global levels; grant recipients and implementing partners at the country level; fund portfolio managers at the Secretariat; technical assistance partners at the country and global levels; civil society representatives. 16 focus countries	Over 900 individuals interviewed
Evaluation of the President’s Emergency Plan for AIDS Relief (PEPFAR) [16]	Assess PEPFAR's performance and its effects on health	Described interviews conducted in terms of 8 roles across countries Total staff estimates were not quantitatively described	Individual source population not described 31 focus countries, 13 of which were visited	383 interviews conducted across the 8 roles and country contexts
Second Gavi Evaluation [17]	Assess the extent to which Gavi met its four strategic goals and the extent to which it has added value at global and country levels	Stakeholders of Gavi; 4 internal roles and 7 external roles described. Total stakeholder estimates were not quantitatively described	Online survey sent to 1,000 stakeholders Manager survey sent to 76 Expanded Program on Immunization managers	282 responses received, including half from partner institutions 23 responses received 109 consultations with roles described

Indeed, there is a gap in methods for defining and implementing a sampling process for participants of a large, complex initiative that could span multiple countries and organizations that exists both in broader biomedical and social sciences [1, 7, 8, 12]. The goal of this paper is to describe a sampling approach for surveying complex and multi-institutional partnerships, and to identify a study population for evaluating a complex program than spans multiple countries and organizations based on a knowledge translation project around the GPEI and polio eradication activities.

### Synthesis and translation of research and innovations from polio eradication

The Synthesis and Translation of Research and Innovations from Polio Eradication (STRIPE) project seeks to map, package and disseminate knowledge gained from the GPEI and polio eradication activities and is described elsewhere [18]. To that end, the project conducted a “tacit knowledge survey” to capture ideas, approaches and experiences that are not documented, but relevant to understanding both intended and unintended results from polio eradication activities. In order to ensure that findings from the tacit knowledge survey represent the full spectrum of actors involved in polio eradication activities, a systematic approach was developed to define the total possible population of actors, list specific individuals that fit within the total population, and then derive a comprehensive sample of actors involved in global polio eradication activities at various levels. This paper describes the development and application of this approach for identifying a population of actors involved in the GPEI, and methods for conducting a tacit knowledge survey assessing lessons learned from the initiative. It is hoped that the steps distilled from completion of this activity will facilitate studies on other complex global health initiatives, and advance efforts to generalize and replicate findings from these studies in future.

## Results

### Defining levels of representation

The implementation of a complex initiative generally has many phases, including identifying stakeholders; designing a program; mobilizing resources; work planning; implementing; and monitoring, reviewing, and evaluating implementation [19]. For polio eradication, a wide range of stakeholders, including governments, country partners, and the five partner organizations of the GPEI (Rotary International, the World Health Organization (WHO), United Nations International Children’s Fund (UNICEF), Centers for Disease Control and Prevention (CDC), and the Bill and Melinda Gates Foundation (BMGF)) participate in various phases of program implementation across numerous operational and organizational levels. The GPEI has operated in over 100 countries since inception in 1988, partnering with various levels of government (including host countries, bilateral/sovereign donor countries, and implementing countries) and with non-government organizations (NGOs) and civil society organizations (CSOs) as dictated by the local context. To derive a comprehensive sample of actors involved in GPEI and other polio eradication activities, information should be collected to reflect at a minimum (i) context typologies for polio implementation; (ii) implementation phases of polio eradication initiatives; and (iii) the pre-identified activities included in these phases.

Since it was not feasible to systematically collect lessons learned from every context where GPEI has operated, seven countries were purposively selected to represent a range of epidemiologic and operational contexts: Afghanistan, Bangladesh, Democratic Republic of Congo (DRC), Ethiopia, India, Indonesia, and Nigeria. The context typologies for polio implementation were defined based on the epidemiological, geographical, income, conflict, and other status^2^ [18]. Relevant organizations were broadly defined to include those involved with funding, researching, designing, managing, and implementing activities that contribute to polio eradication. In certain cases, this process was aided by referring to a country-specific “Polio Program Summary” which described the landscape of polio eradication in a country. After identifying these organizations and levels, common operational goals were identified through reviews of GPEI strategic plans through the lens of implementation frameworks [20, 21]. This information was collated in an operating list of contexts, organizational levels, and operational goals which was the starting point for describing the universe of individuals potentially involved in GPEI and polio eradication activities (Table 3).

**Table 3:** Levels of representation within polio eradication

Country Polio Epidemiologic Contexts as of 2018 (and STRIPE focus countries)	Examples of Organizational and Operational Levels	Polio Goals
Polio Endemic: • Afghanistan • Nigeria Outbreak: • DRC High risk: • Ethiopia^a^ Polio free: • Bangladesh • India • Indonesia^a^	• Core GPEI members ○ BMGF, CDC, Rotary, UNICEF, WHO ○ Regional polio surveillance laboratories • Country government officials ○ State, District, and local level government ○ Subnational monitoring boards ○ Vaccinators • Civil Sociey Organizations ○ International Non-Government Organizations ○ Local Non-Government Organizations ○ Community organizers ○ Local and religious leaders • Other donor organizations and development partners ○ International development partners (USAID, DFID, etc) ○ Local donors/development partners	• Resource mobilization • Partnership/alliance development • Strategy development and planning • Strengthening delivery services • Vaccination • Surveillance • Community Engagement • Monitoring and Evaluation

^a^Ethiopia was “high risk” and Indonesia was “polio free” as designated by GPEI at the time of STRIPE protocol preparation. Both countries have since reported cases of circulating vaccine-derived poliovirus and are classified as “outbreak” countries as of 27 October 2019

### Theorizing a target population: the polio universe

A universe is a tool for understanding all units that may be involved in a study, and in the STRIPE context refers to all potential individuals that qualify for inclusion in the tacit knowledge survey [6]. In addition to determining the individuals eligible for the study, defining the polio universe is also an essential step for understanding the population to which the results of the study will be applicable. The polio universe across the study areas was considered to be “the population of individuals who have been directly involved in implementing polio eradication related activities for 12 or more continuous months between 1988 till date.” Implementing activities refer to all cycles of implementation, including GPEI-related funding, policy, programming, and research cycles. Researchers in the seven partner countries modified the parameters surrounding this definition to best represent the realities of polio eradication activities in their own setting as described below (Table 4).

**Table 4:** Polio universe, source population, and study populations

Country	Polio Universe Description	Estimated Universe Size (Target Population)	Operationalizing the Polio Universe	Proposed Sample (Source population)	Contact Plan	Study population
Afghanistan	Individuals with knowledge and expertise of polio programs in the country from directly working in the polio eradication program. This included anyone who has worked directly in any level for policy formulation, policy implementation, program management, and field operation. To have an inclusive sample, Afghanistan’s Polio Universe was based on two frameworks; WHO health system building blocks and Afghanistan National Polio Eradication Initiative.	185,041	All national actors interviewed with online survey (206 identified) All provincial EPI teams contacted with mobile phone survey (442 identified) Purposively selected 7 provinces for in-person interviews	65,041	Online survey (31 respondents, 15.0% response rate) Mobile Phone survey (126 respondents, 28.5% response rate) In-person interviews (365 respondents)	522
Bangladesh	Individuals who spent 12 or more continuous months working directly in implementing activities under the polio eradication program of Bangladesh between 1988 till date.	51,500	Snowball sampling to identify respondents due to lack of program intensity	140	Online survey (23 respondents) In-person interviews (83 respondents)	106
DRC	Since Polio was integrated within the EPI Activities at the operational level, the Polio universe comprised all those dealing with it at different levels of the health pyramid, including external technical partners (such as WHO, UNICEF, Sabin Institute) and funding agencies (such as the World Bank, the BMGF, Rotary International, etc.)	300,000	Stratified health districts based on immunization coverage (good vs bad), history of polio epidemics, presence of AFP cases, and history of cVDPV outbreaks and randomly sampled districts by context type Randomly sampled health areas within each district and then interviewed individuals within the health areas.	85,000	Online survey (136 respondents, 34.75% response rate) In-person interviews (400 respondents)	536
Ethiopia	The polio universe in Ethiopia included all individuals and partners who have been involved for 12 or more continuous months in implementing polio eradication activities in the country at national and/or sub-national levels (Region, Zone, District and health facilities); in public and/or private sector; and for NGOs between 1996 and 2018. It included individuals working at the national level (FMoH, national agencies such as EPHI and PSA, GPEI partners, NGOs, Professional Associations), Universities and Research Institutes, Regional Health Offices, Zonal and District Health Offices, Hospitals, health centers, health posts, public and private health facilities, religious and clan leaders, and communities and volunteers.	100,000 health workers who could have been involved in polio eradication	Purposively selected 5 regions (Addis Ababa, Benishangul-Gumuz, Gambella, Oromia, and Somali)	150	Online survey (2 respondents, 6.8% response rate) In-person interviews (106 respondents)	108
India	The polio universe consisted of persons from Global Polio Eradication Initiative (GPEI) partner organizations, permanent and contractual employees of central and state government, members of national and international NGOs, teaching and non-teaching staffs of schools, medical and non-medical staff of different at levels of government hospitals and frontline health workers, media persons and volunteers. In addition, influencers from religious institutions like temples, mosques, church etc.	Estimated over 2.4 million people were involved in polio eradication	Stakeholder consultation workshop with high level actors enabled a list of 4,957 contacts	4,957	Online survey (352 respondents 7.1% response rate) In-person interviews (165 respondents)	517
Indonesia	Actors who have spent 12 or more continuous months directly and indirectly for polio activities at national and sub-national level (provinces and districts) and restricted to time period between 1995-2014 (polio free certification)	103,252	Purposively selected six provinces (Aceh, Banten, West Java, DI Yogyakarta, East Java, East Nusa Tengarra)	32,502	Online survey In-person interviews	322
Nigeria	A census of all actors who were involved in GPEI activities in Nigeria, from high level planners to project managers and field immunizers on a long-term basis (12 or more continuous months) within well-defined periods between 1988 and 2018. Partners that were involved in the survey include CDC, Rotary, WHO, UNICEF, NSTOP, NEOC, NPHCDA, Federal Ministry of Health, State Ministry of Health and Local Government Area level.	14,064	Purposively selected 10 states (Anambra, Bayelsa, Borno, FCT, Kano, Lagos, Nassarawa, Ondo, Oyo and Sokoto). Randomly selected 4 Local Government Areas from each state	3,906	Online survey (793 respondents) In-person interviews (160 respondents)	953
Global Survey	A population of individuals who have been directly involved in implementing activities under the GPEI between 1988 till date. Implementing activities refers to all cycles of implementation, including GPEI-related funding, policy, programming, and research cycles. The population includes individuals who have spent 12 or more continuous months working on activities under the GPEI between 1988 till date.	3,929 core staff active at the global level	Distributing the survey to all GPEI sub-groups Gatekeepers from each organization to either provide contact information or distribute the survey within global organizations GPEI listservs referring individuals to a webpage with the survey	1,400	Online survey (891 respondents)	891
*Definitions and Totals*	*Across 7 countries and global staff dedicated to polio eradication from 1988 to date, the estimated polio universe:*	*3,157,786*	*Across 7 countries and the global staff dedicated to polio eradication, the number of individuals who could be reached to take the survey:*	*193,096*	*Number of respondents included in the study population*	*3,955*

While the core aspect of the polio universe definition did not change, i.e. you have to have worked for a continuous 12 months, country teams restricted the period considered to better match the reality of polio operations within their country. For example, Indonesia’s last polio case was in 2006 and the polio-free certification was given in 2014, thus the country team prioritized data collection between 2000-2015 to match the period that polio activities became established in their country. Adapting the time frame of the polio universe is necessary for all countries to ensure that data was collected from the most relevant actors in a specific context. All country teams identified the organizational levels within a country and then estimated their polio eradication-specific workforces over the relevant period of time. Afghanistan and DRC were two countries where additional information based on the structure of their health systems was combined with the organizational levels for polio program to describe a polio universe.

In Afghanistan, where NGOs play a large role in the health system, the polio universe was defined in terms of both state and non-state actors. Thus, the Afghanistan polio universe was systematically described along the six WHO health systems building blocks (service delivery, human resources, medical supplies, governance, health information, and finance) and stratified by government and non-government actors for each building block [22]. This framework-based approach helps to systematically describe the types of actors in an otherwise fragmented health system. In contrast, in the DRC, polio eradication closely follows the national health system, as it is integrated within the Expanded Program on Immunization (EPI) activities. Members of the polio universe were classified accordingly, including actors involved in immunization at the various health system levels, financing, technical, and implementing partners operational in the DRC. From this more concretely derived polio universe, it was then possible to utilize probability-based sampling methods.

### Enumerating the source population

While describing the polio universe is necessary to understand all potential individuals who are eligible for responding to the study, it is not possible to implement data collection based on inclusion in the polio universe alone. The source population refers to all those who belong in the polio universe and can be reached for a tacit knowledge survey. Strategies for identifying specific individuals within a polio universe were shared and adapted across country teams to form a country-specific source population. One strategy involved identifying key government agencies, NGOs, religious organizations, civil society organizations, and academic communities involved in GPEI activities in the country in question through a variety of sources. Another strategy involved contacting point persons at identified key organizations and requesting lists of contact information for people and organizations who may belong to the polio universe across various countries. Once polio universes were defined and described, country partners used different criteria and assumptions for estimating the sample size taking into consideration the operational feasibility of the sample.

Most countries identified universes that contained more than 100,000 individuals and were forced to focus data collection efforts on key populations to achieve subnational representation of the polio universe. The most common method used was the selection of subnational geographic units for increased sampling. India’s efforts to narrow down the largest estimated universe began with a stakeholder consultation workshop and resulted in the generation of a sampling frame of 4,792 individual respondents, complete with contact information. Review of the actors on this list determined that while state and national level actors from a range of organizations were included, frontline workers, such as Accredited Social Health Activists and Auxiliary Nurse Midwives were not represented because access to the internet was limited or their contact information was not available. Thus, a plan was made to perform snowball sampling to find frontline workers in four high-risk States in India.

In Ethiopia, an at-risk country at the time of study design, the polio universe was estimated at 100,000 individuals by contacting key government agencies such as ministry of health, regional health bureaus, agencies, health professional associations, GPEI partners, civil society organizations, and international and local NGOs and obtaining estimates of their staffing levels. The most at-risk areas in the country, especially Somali, Gambella, and Benishangul Gumuz States, and southern Oromia State which borders Kenya were selected based on their highest risk for polio transmission and intense activities from the government and partners. Overall, a list of 150 key individuals at national, regional, zonal, district and health facility levels including frontline health workers was compiled with complete contact information for administering the survey.

In countries that had been free from wild poliovirus for over a decade, such as Bangladesh and Indonesia which last reported cases of wild polio in 2006, core actors were readily identifiable, however large population samples of actors were harder to identify. After attempting other sampling options, the Bangladesh team used a strategy of strict snowball sampling, starting from GPEI core partners and national health officials to identify 140 individuals across the country who were involved in polio eradication efforts and could be reached by survey efforts. Indonesia’s disperse geography necessitated a different approach. Six provinces were selected, in consultation with the Ministry of Health, to conduct snowball sampling to obtain a sampling frame of subnational respondents. These provinces were based on areas of intense government activities where important lessons could be learned, such as the first province to switch to inactivated polio vaccine, the locations of the last wild poliovirus outbreak, and areas of conflict.

### Obtaining a comprehensive sample

Data from the polio eradication-specific workforce was collected by administering a survey to a sample of the enumerated source population. Data collection involved a globally-distributed online survey and seven concurrent surveys implemented at the national and subnational levels by partner institutions in seven focus countries. The survey was administered to those identified in the source population, and those who answered comprise the study population. Respondents were provided with the opportunity to remain anonymous to ensure that they could voice their opinions without fear of repercussions. Several challenges prevented study teams from reaching the entire enumerated populations, including inaccurate contact information, individuals changing roles or leaving the country, and/or relatively low response rates. To address these challenges, teams utilized various methods of administering the same survey tool to ensure that responses were characteristic of the enumerated source populations both in terms of quantity of responses as well as a variety of respondents. The first method for reaching individuals was through an online survey which was sent directly to the emails of enumerated participants and made available in English and in eight languages commonly used in the partner countries. Challenges specific to this method included the fact that potential respondents may have missed the email, the email may have gone to ‘spam’ folders, or respondents may not have been aware of the purpose or legitimacy of the survey. Other methods, including in-person and mobile phone administration of the survey, supplemented these efforts and were useful in reaching enumerated respondents who were not reachable through online methods. Most countries utilized a combination of distribution techniques for administering the survey to a population that would represent a variety of organizational levels. The best method of reaching international, national, and some state-level actors was generally recognized to be an online survey (available in English and locally relevant languages), while in-person interviews were employed particularly to reach front-line immunizers. All methods utilized the same survey questionnaire (made available elsewhere in this series, see [18]), however questionnaires administered to field workers were translated into more accessible, locally appropriate terminologies.

Where countries had constrained their polio universe to a narrower source population that included a complete sampling frame, it is possible to evaluate the response rate^3^ of the various survey distribution methodologies. In Ethiopia, the online survey platform had the smallest observed response rate, as only 6.8% of identified actors responded to the survey. Most countries experienced similarly low response rates to the online survey, however DRC’s online survey had a relatively high response rate of 34.8% (Table 4). Close relationships between the study team and the government’s vaccination program could account for such high levels of buy-in in DRC. All respondents who were administered the survey in-person consented and participated in the survey.

In areas where polio activities were ongoing, several strategies were effectively used to increase the study population. Nigeria was able to obtain a study population of 953 respondents by leveraging existing networks. The team utilized connections within the University of Ibadan College of Medicine to distribute the survey to former Master's students who had been involved in polio work, which, in concert with more available internet connection, contributed to the far reach of the survey. In contexts with conflict and less reliable internet connection, online surveys had lower response rates and in-person interviews were not feasible. Afghanistan increased its study population by administering the survey via mobile phone for respondents located in conflict areas. While Afghanistan was the only country to utilize this method, the response rate for the phone interview was nearly twice as high as the online survey (Table 4).

### Reflecting on the obtained sample

It is important to reflect on the processes used and the samples obtained to provide insight on the strength of conclusions drawn during subsequent analysis. For the combined tacit knowledge survey, a total of 3.16 million individuals were estimated to exist in the polio universe, of which information was obtained for 193,096 individuals (about 6% of the universe). A total of 3,955 individuals were reached with the survey, equaling about 2% of the source population.

Recognizing that various challenges in countries (e.g. length of time since polio eradication in a geographic area, reaching conflict affected areas, reaching hard-to-reach areas, and ensuring representation of front-line workers) required diversions from purely probability-based approaches, a number of strategies were adopted to obtain perspectives from relevant polio actors. For example, the sheer size of India’s polio program complicated the estimation of a polio universe, resulting in a rough estimate of some 2.4 million individuals. In Bangladesh, actors involved in polio were harder to find due to the length of time since eradication, which necessitated the use of snowball sampling. Conversely, the structured organization of DRC’s polio universe allowed the team to confidently estimate the number of individuals in their polio universe and utilize systematic random sampling methods, increasing the likelihood that their study population is indeed representative of their polio universe. Variability in the process of operationalizing the polio universe, defining a source population, and data collection limits the strict generalizability of the findings of specific country surveys but yields important conclusions for the global polio eradication efforts more broadly. Across all surveys, efforts were made to specifically seek out and collect data from front-line workers, ensuring that those with first-hand experience were included across settings. The strategies and processes utilized in the tacit knowledge survey can be distilled into general steps for sampling from a complex initiative.

### Generalizing to other complex initiatives

Based on the systematic approach used to obtain a sample for lessons learned in GPEI, the steps for obtaining a comprehensive sample for studying complex initiatives can be summarized as follows;

**Table:** 

(i) State research goal(s)	
(ii) Describe the program of interest	
(iii) Define a sampling universe to meet these criteria	
(iv) Estimate the size of the sampling universe	
(v) Enumerate a source population within the universe that can be feasibly reached for sampling	
(vi) Sample from your source population (collect data)	
(vii) Reflect on the process to determine strength of inferences drawn	

The STRIPE project demonstrated that the methodology described above can be effective in obtaining an extensive sample of participants in a complex global health initiative. The project first defined its goals to map and synthesize tacit knowledge, ideas, approaches, and experiences that are not documented but are relevant to understanding both intended and unintended results from polio eradication activities within various contexts. The second step was then to describe efforts that have and continue to contribute to the eradication of polio; including generalizable individual-level activities and goals, organizational and operational levels, and contexts/countries in which they operate.

In order to obtain the desired results, the STRIPE project collected responses from individuals associated with organizations involved with funding, researching, designing, managing, and/or implementing activities that contributed to polio eradication. A definition for a universe containing all units related to this objective was defined and adapted to better meet specific contexts. Two common approaches were to: (i) conservatively estimate the number of all individuals who could have possibly been involved using health-worker level estimates (Ethiopia and India) or (ii) estimate the workforce of key organizations identified (Bangladesh and Nigeria). Next, individuals within the universe were enumerated by a number of strategies, including primarily: (i) purposively or randomly selecting geographical sub-units within a country for enumeration (Afghanistan, DRC, Ethiopia, Indonesia, and Nigeria); (ii) utilizing snowball sampling when polio activities were further removed from time (Bangladesh) or to include perspectives of frontline workers (Indonesia, India, and Ethiopia); and (iii) convening stakeholders and enumerating as many individuals as possible within key organizations (India). Next, procedures were implemented to obtain a comprehensive sample of the enumerated population. Approaches utilized include: (i) snowball sampling of frontline workers within study areas (Bangladesh, Ethiopia, and Indonesia); (ii) random sampling within each identified study area (DRC); (iii) distributing to the census of the source population (India); and (iv) using existing networks to distribute the survey (Nigeria and Afghanistan). Finally, survey respondents and response rates can be compared with original estimates of the source population and universe to assess the comprehensiveness of the survey.

This sampling approach has several benefits that complement existing methods. Primarily, this systematic approach maps individuals involved in a complex initiative across operational levels and contexts, ensuring that perspectives are collected from actors who have a wide range of experiences. Such a methodology allows for the comparison of tacit knowledge both vertically (from frontline workers to strategic planners) and horizontally (across organizations and countries), enabling a holistic and comprehensive approach to synthesizing lessons learned. Such approaches are essential in complex initiatives and partnerships, which have historically focused on lessons learned from a limited number of perspectives, especially from policymakers and managers at global and national stages, leaving a gap in our understanding of lessons from the perspectives of sub-national and community level actors. This is the experience to date in the large-scale evaluation of polio eradication activities. Since 2011, the Independent Monitoring Board (IMB) has been supporting the GPEI in its aims to interrupt polio transmission globally. Comprised of experts acting independently of GPEI, the IMB has published 17 reports on the status of polio eradication and recommendations to accelerate eradication as of November 2019 [23]. The IMB largely relies on data provided by GPEI organizations, as well as conversations with GPEI and government organizations, and short field visits [24, 25]. The STRIPE project collects much of the same data as the IMB; with reports and data provided by GPEI organizations captured in the series of literature reviews and conversations with policymakers captured in key informant interviews [18]. While the IMB has recommended the collection and review of field-level perspectives since its earliest days, there has yet to be a systematic collection of perspectives across the entire range of actors involved in polio eradication [25]. The main contribution of the tacit knowledge survey is to fill this gap in knowledge, and provide perspectives and experiences across a range of actors, organizational, and operational contexts that have not systematically been compared and synthesized to date.

This sampling approach will yield comprehensive data that will contribute new lessons learned to the polio eradication narrative, however there are some limitations that should be acknowledged. Operational obstacles prevented the survey from being implemented as planned, including the inability to obtain up to date and accurate contact information for potential respondents at all levels and inaccess to key operational areas, necessitating the adoption of multiple data collection approaches. Challenges also existed in the potential introduction of systematic error in data collection and entry across teams, as a variety of methods were used for data collection (e.g. self-report for global respondents, in-person collection by an enumerator via tablets, and separate data entry by data managers in some settings), however constant contact between study teams and a central, online platform for data collection helped to ensure standardization. Future attempts to derive lessons from complex global health initiative could be based on study populations that are defined in real-time, alongside the program implementation activities at various levels – which may not necessarily yield a consistent approach across settings but will be better aligned to the initiative.

As mentioned previously, differences in country team approaches result in varying levels of generalizability of findings from country-level surveys to holistic country polio eradication efforts. Some groups or countries may be over-represented (e.g. Nigeria’s relatively large study population) and others may be under-represented (e.g. Bangladesh or Ethiopia’s relatively small study population), either as a result of gaps in theorizing the entire universe of actors involved, missing data when operationalizing their universe into a source population, or inability to reach individuals during data collection. However, in all contexts, key actors were readily found and included in the survey, and additional efforts were made to engage frontline workers, ensuring that the overall study population is indicative of experiences related to global polio eradication more broadly. Moreover, final conclusions about polio eradication under this study will not rely solely the data from the survey, but will be derived by combining data from multiple research streams (including literature reviews, key informant interviews, and health system analyses), contributing to the strength of these generalizations.

## Conclusion

This paper provides a broad methodology for describing, enumerating and sampling from participants of a complex population and provides examples of methodological adaptations to best fit the application to distinct contexts. Solutions presented in this paper fill a gap in current operational, implementation, and health systems research, in that there are currently few methods for systematically sampling across complex public health initiatives that are implemented internationally by several organizations and stakeholders. By applying these methods, the STRIPE tacit knowledge survey was able to describe the universe of actors involved in GPEI, obtain information about these actors, and sample an impressive 3,955 individuals from the initiative. Such a systematic and extensive approach to collecting lessons learned from a global health initiative has not previously been undertaken. The insights obtained from the tacit knowledge surveys can be generalized to the overall polio eradication effort, as considerations were made at several levels to ensure rigor and comprehensiveness. Potential applications of the data collected in the STRIPE tacit knowledge survey can include investigations into country-specific barriers and facilitators of program success, analysis of common barriers and facilitators experienced over time, and a comparison of findings with IMB recommendations. Future research activities can draw from these methodologies to inform further systematic health systems research and increase the efficacy of public health initiatives by translating knowledge gained into improved programming that saves lives.

## Data Availability

The datasets used generated from the current study are available from the corresponding author on reasonable request and will be made available at the STRIPE repository upon project completion.

## Abbreviations

BMGF: Bill and Melinda Gates Foundation
CDC: United States Centers for Disease Control and Prevention
CSO: Civil Society Organization
DFID: Department for International Development
DRC: Democratic Republic of the Congo
EPI: Expanded Program on Immunization
GPEI: Global Polio Eradication Initiative
IMB: Independent Monitoring Board
NGO: Non-Government Organization
PEPFAR: US President’s Emergency Plan for AIDS Relief
PMI: President’s Malaria Initiative
STRIPE: Synthesis and Translation of Research and Innovations from Polio Eradication
UNICEF: United Nations Children’s Fund
USAID: United States Agency for International Development
WHO: World Health Organization

## Footnotes

1 In March 2019, a sixth partner, Gavi the Vaccine Alliance, has joined the GPEI core partnership. Since Gavi was not a core partner during protocol development and data collection, this paper does not include the organization as a core member

2 In addition to polio status classification, the focus countries were selected based on the diversity and intensity of GPEI activities, informed by the extent of GPEI resources invested in that country over the last two decades. Under the assumption that knowledge assets around polio eradication will be concentrated where resources have been heavily targeted. The focus countries were further selected to represent different geographical regions where polio eradication activities have ramped up in recent times, country income classifications, conflict-affected compared to stable countries, and countries that could serve as influential regional leaders to facilitate uptake of knowledge by other countries in their region (STRIPE protocol paper)

3 Response rates are calculated as the number who responded to the survey / the number who were asked to respond to the survey.
